# Prior Knowledge of Target Direction and Intended Movement Selection Improves Indirect Reaching Movement Decoding

**DOI:** 10.1155/2017/2182843

**Published:** 2017-04-13

**Authors:** Hongbao Li, Yaoyao Hao, Shaomin Zhang, Yiwen Wang, Weidong Chen, Xiaoxiang Zheng

**Affiliations:** ^1^Qiushi Academy for Advanced Studies, Zhejiang University, Hangzhou 310027, China; ^2^Department of Biomedical Engineering, Zhejiang University, Hangzhou 310027, China; ^3^Key Laboratory of Biomedical Engineering of Ministry of Education, Zhejiang University, Hangzhou 310027, China; ^4^Zhejiang Provincial Key Laboratory of Cardio-Cerebral Vascular Detection Technology and Medicinal Effectiveness Appraisal, Hangzhou 310027, China

## Abstract

*Objective.* Previous studies have demonstrated that target direction information presented by the dorsal premotor cortex (PMd) during movement planning could be incorporated into neural decoder for achieving better decoding performance. It is still unknown whether the neural decoder combined with only target direction could work in more complex tasks where obstacles impeded direct reaching paths. *Methods.* In this study, spike activities were collected from the PMd of two monkeys when performing a delayed obstacle-avoidance task. We examined how target direction and intended movement selection were encoded in neuron population activities of the PMd during movement planning. The decoding performances of movement trajectory were compared for three neural decoders with no prior knowledge, or only target direction, or both target direction and intended movement selection integrated into a mixture of trajectory model (MTM). *Results.* We found that not only target direction but also intended movement selection was presented in neural activities of the PMd during movement planning. It was further confirmed by quantitative analysis. Combined with prior knowledge, the trajectory decoder achieved the best performance among three decoders. *Conclusion.* Recruiting prior knowledge about target direction and intended movement selection extracted from the PMd could enhance the decoding performance of hand trajectory in indirect reaching movement.

## 1. Introduction

Brain-machine interfaces (BMIs) develop a direct pathway between the brain and external devices, which aims to help amputees or paralysis patients regain their movement functions [1, 2]. The decoding method is the essential part of BMIs which maps the neural activities to movement trajectories. Numerous decoding methods have been proposed in recent decades, such as state-space model [3, 4], artificial neural networks [5], and reinforcement learning [6, 7], which have been applied in many BMIs successfully, such as a robot arm [8–10] and computer cursor trajectory estimation in two and three dimensionality [11, 12]. In most studies, the task is point-to-point target-oriented center out or variant center out [13–18], in which the target direction and initial movement direction are correlated.

However, the environment of daily life is more complex. For example, obstacles between food and human beings would make fetching trajectories curved to avoid running into it. Such cases challenge the performance of decoding methods with the decoupled target direction and initial movement direction. The study of the complex task could push the limits of BMIs and accelerate the clinical translation [19]. Actually, researchers have designed tasks to dissociate the target direction from initial movement direction, such as curved movements [20], environment with specific paths [19], or obstacles [2]. However, most of the decoding methods were applied to the point-to-point target-oriented tasks. Work needs to be done to extend the proposed methods to more complex tasks, which could extend the performance limits of BMIs.

Multiple cortices are involved corporately and hierarchically to process the complex tasks. The primary motor cortex (M1) plays the role of passing neural impulses down to the spinal cord and controlling the execution of movement. The dorsal premotor cortex (PMd) is responsible for higher-level movement control, including movement preparation, sensory and spatial guidance of reaching, or some direct control of reaching movement [21–23]. Planning could happen before movement onset, and a delay epoch can contribute to mature performance. In 2012, Pearce and Moran designed an obstacle-avoidance task in which the initial movement direction was confined to induce a curved center-out task, and they found that population vectors (PVs) [15] of one monkey point to the target at first and then turn to the movement direction with relevant visual cues showing up during the delay epoch [2]. In fact, target direction and initial movement direction are two key instructions to finish the indirect reaching. In 2007, Yu et al. extracted target direction during planning as a prior information for the following trajectory estimation [16]. In 2013, Shanechi et al. estimated the target information from the PMd and SMA before movement initiation to improve the trajectory decoding in a center-out task [18]. Similarly, the movement direction could be estimated and integrated to improve the trajectory decoding based on the finds of Pearce and Moran [2].

Several methods have been proposed to decode the indirect reaching movement task [3, 24, 25]. In 2012, Gilja et al. proposed the recalibrated feedback intention-trained Kalman filter (ReFIT-KF) to improve the online decoding performance of target-oriented reaching movement task [3]. Researchers also applied ReFIT-KF on the obstacle-avoidance task with promising performance. However, the design of ReFIT-KF did not consider the properties of indirect reaching movement and the obstacle-avoidance performance benefitted from the visual feedback and modulation of neural activities. So the algorithm would not work well in an offline case. Similarly in 2017, Shanechi et al. enhanced the online reaching movement control by rapid control and feedback rates [24]. They applied this method to the obstacle-avoidance task. However, the same issue exists as ReFIT-KF. In our previous studies, the correntropy-based attention-gated reinforcement learning (CAGREL) was proposed to decode the obstacle-avoidance task by setting a secondary target to avoid the obstacle manually [25]. For obstacle-avoidance task, more kinematics parameters are involved, so an algorithm framework that integrates different information is needed [26]. In 2007, Yu et al. built the mixture of trajectory models (MTM) based on recursive Bayesian estimation (RBE) [18, 27–32] for neural decoding of goal-directed movements [16]. They combined several trajectory models, each of which was more accurate within the limited regime of movement (trajectory to one specific target), with probabilistic weights predicted by planning activities. The probability of target direction was estimated from the PMd during the planning period. However, for a more complex task such as obstacle-avoidance task, it is still unknown whether the neural decoder combined with only target direction information could work.

In this study, we examined how target direction and intended movement selection were encoded in neuron population activities and tried to improve the indirect decoding by integrating more prior knowledge. Two monkeys were trained to perform delayed obstacle-avoidance task. One Utah array was implanted in the PMd area for each monkey. Population vector (PV) and principal component analysis (PCA) were utilized to analyze neuron encoding properties during planning epoch. For comparison of movement trajectory estimations among decoders with no prior knowledge, only target direction and both target direction and intended movement selection were carried out.

## 2. Experiments and Methods

### 2.1. System Setup and Training Tasks

**Figure 1:**
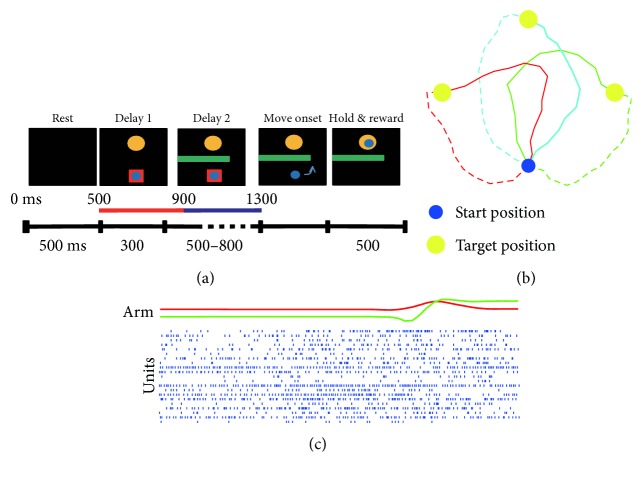
Delayed obstacle-avoidance task and data acquisition. (a) Task epochs (top) and timeline (bottom). The red square (length of side: 130 pixels) in task epochs indicates delay cue, during which monkeys had to hold at the start position. The green bar represents an obstacle, touching of which would result in trial failure. The small blue ball and big yellow ball represent the moving cursor and target, respectively. The red and blue bars above the timeline show the intervals used for target direction and intended movement selection decoding, respectively. (b) Averaged reaching trajectories from the start position (small blue ball) to target position (big yellow ball) in one representative session (B140530). Depending on the position of the obstacle (not shown), there were two trajectories to the same target represented by solid and dashed lines in the same color. One trial would be started from any one of the four positions (top, down, left, and right), and the target ball would be on one of the rest three positions randomly. (c) Simultaneously recorded hand positions (X position, red; Y position, green) and spike trains in one representative trial.

In this study, two male rhesus monkeys (*Macaca mulatta*, labeled as monkeys B and C) were trained to perform a delayed obstacle-avoidance task using their upper limbs (right upper limb for monkey B and left upper limb for monkey C). In the task, monkeys were seated in a primate chair and one monitor was placed 50 cm away in front vertically. As shown in Figure 1(a), the monkey was trained to manage a 2D manipulator (20 × 20 cm range) to move a computer cursor (small blue ball) from the start position to the target (big yellow ball) without touching the obstacle (green bar) to get a water reward.

The target position could appear on the left, top, and right with the start position on the bottom, as shown in Figure 1(b). The average trajectories across 20 trials were shown. And trajectories to the same target were labeled by the same color. The bold cyan trajectory was the case shown in Figure 1(a). There were six trajectories with a fixed start position. Additionally, the cases that the start position was located at the left, top, and right were also collected. Generally, the target position in the current trial was the start position for the next. Sometimes, monkeys moved the cursor away from the start positions during rest, and those cases were discarded in our study. In total, there would be 24 (6 trajectories × 4 start positions) conditions where data were collected. This task partly simulated the complex environment by adding an obstacle between the start position and target position, which challenged the performance of decoding methods.

Specifically, the task started with the appearance of the computer cursor and target, as illustrated in Figure 1(a). The cursor was located at the bottom and surrounded by a red square, indicating that monkeys had to hold at this start position. And the target was located at the top. This epoch would last for 300 ms (delay 1) for monkeys to acquire the information of target direction. Then the obstacle appeared and lasted for another randomized time (uniform distribution from 500 ms to 800 ms, delay 2), which was for monkey planning to avoid the obstacle. The disappearance of the red square signaled the go cue. Monkeys moved the cursor from the bottom to top in a curved trajectory to avoid the obstacle. Monkeys were required to hold at the target position for 500 ms to get a liquid reward. A rest time of 500 ms was set between two trials.

### 2.2. Surgery Procedures and Data Acquisition

Neural data were collected by a 96-channel microelectrode array (arranged in a 10 × 10 matrix, 4.2 × 4.2 mm, Blackrock Microsystems, Salt Lake City, UT, USA) [33] implanted in the contralateral PMd for both monkeys. Additionally, two head posts were fixed on the skull with titanium screws to stabilize head and array pedestal during neural recording [34]. The surgery was performed under general anesthesia induced by ketamine (10 mg/kg) and diazepam (1 mg/kg). A deep anesthesia was induced by endotracheal administration of isoflurane (1%-2%) with veterinary anesthesia ventilator (Matrx VME2, Midmark, Orchard Park, NY, USA) during the surgery. The vital signs were monitored by a physiological monitor. Body temperature was maintained by a heating pad (T/PUMP, Gaymar, Orchard Park, NY, USA). Craniotomy was performed over the premotor cortex, and the dura was incised to place the array. The array was quickly inserted into the cortex by a pneumatic insertion device (Micro Implantable Systems, Salt Lake City, UT, USA). The surgical procedure was detailed previously in [5]. After the surgery, the antibiotic therapy lasted for 5 days and monkeys had at least one week to recover. All procedures were approved by the Animal Care Committee at Zhejiang University, strictly complying with the Guide for Care and Use of Laboratory Animals (China Ministry of Health).

Neural activities acquired by the array were transmitted to Cerebus data acquisition system (Blackrock Microsystems, Salt Lake City, UT, USA). Analog waveforms were amplified, band-pass filtered (Butterworth, from 0.3 Hz to 7.5 kHz), digitized (16-bit resolution and 30 kHz sampling rate), and high pass filtered (Butterworth, 250 Hz). The spikes were detected using a thresholding method (at a level of −4.5 times root mean square of baseline) and sorted by commercial software (Offline Sorter, Plexon Inc., Dallas, TX, USA). Trajectories of manipulator and epochs of the task were recorded simultaneously with neural signals, as shown in Figure 1(c). Ten data sessions (from ten different days) have been collected (five for monkey B and five for monkey C). The spikes were binned in 100 ms time scale to predict the prior knowledge and the following trajectory.

### 2.3. Mixture of Trajectory Model

To decode the continuous hand trajectory accurately, we employed a mixture of trajectory models (MTM), which is based on recursive Bayesian methods, developed by Yu et al. [16]. Recursive Bayesian methods need a statistic model of hand trajectories for training, while the MTM probabilistically subdivides the whole trajectories into a limited regime of movement, which could maximally optimize the decoding model in the specific regime. The idea was fitting well with our experiment, in which there were three possible targets for each start point and two possible intended movement selection for each target. This would result in 3 subregimes for targets and 2 subregimes for intended movement selection. According to the MTM framework, the decoding accuracy would be boosted if the information about the regimes were known or partly known. We utilized Bayes' method to obtain the possible targets and intended movement selection during the planning epoch, which will be introduced in the next section. In this section, we will describe the MTM with recursive Bayesian estimation framework included.

The particular probabilistic model in our study referred to that in Kalman filter [4], where kinematics accord with random walk model and observation model is Gaussian [31], which are formulated as follows:

(1) $$\begin{array}{c} \begin{array}{c} x_{t}\left|x_{t-1}\right.,m\sim N\left(A_{m}x_{t-1}+b_{m},Q_{m}\right),\\ y_{t}\left|x_{t}\right.,m\sim N\left(H_{m}x_{t}+p_{m},V_{m}\right), \end{array} \end{array}$$

where *m* ∈ {1,…, *M*} is the label of movement regimes. Taking target direction and initial movement selection into account, there are six movement regimes. Considering target direction only, the number is three. **x**_*t*_ ∈ *R*^2×1^ represents hand position at time step *t* ∈ {1,…, *T*}. **y**_*t*_ ∈ *R*^*n*×1^ represents the neural activities at time step *t*, where *n* represents the number of single units. The state transition matrix **A**_*m*_ ∈ *R*^2×2^, observation transition matrix **H**_*m*_ ∈ *R*^*n*×2^, variance matrix **Q**_*m*_ ∈ *R*^2×2^ and **V**_*m*_ ∈ *R*^*n*×*n*^, and bias **b**_*m*_ ∈ *R*^2×1^ and **p**_*m*_ ∈ *R*^*n*×1^ were all fit to training data and remained consistent during the test.

**Figure 2:**
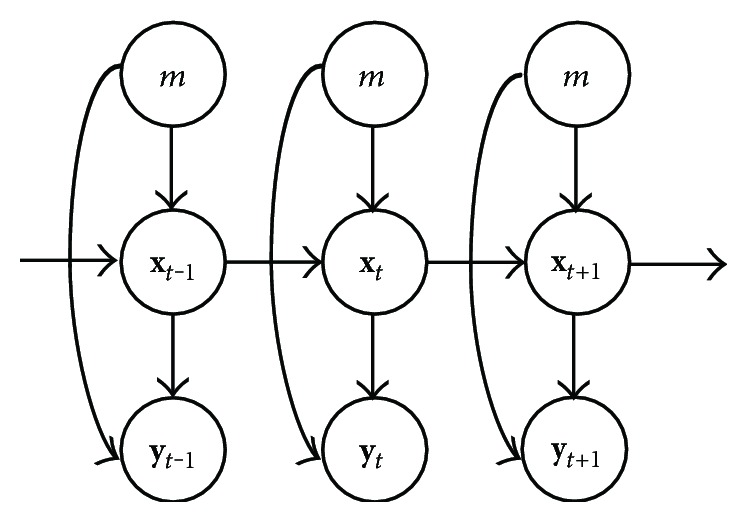
Graphic model for MTM. *m* represents specific movement regime. **x**_*t*_ and **y**_*t*_ represent hand positions and neural activities at time *t*, respectively. Arrows mean dependency relationship between parameters.

The dependency relationships between movement regimes, kinematics, and neural activities are shown by the graphic model in Figure 2. Both of the kinematics and neural activities depend on the movement regimes. Neural activities depend on the kinematics by the observation model and kinematics conform to the random walk model.

Based on the graphic model and Bayes' method, the kinematic estimation at time step *t* is equal to the posterior distribution of hand position **x**_*t*_ conditioned on neural activities from the initial time step to current time step *t*, which is defined as *P*(**x**_*t*_{**y**}_1_^*t*^). To expand the posterior term conditioned on movement regime *m*, we get

(2) $$\begin{array}{c} P\left(x_{t}\right.\left|{\left\{y\right\}}_{1}^{t}\right)=\overset{M}{\underset{m=1}{\sum }}P\left(x_{t}\right.\left|{\left\{y\right\}}_{1}^{t},m\right)P\left(m\right.\left|{\left\{y\right\}}_{1}^{t}\right), \end{array}$$

where *P*(*m*|{**y**}_1_^*t*^) means the probability of movement regime *m* given the neural activities from the beginning to current time step. Furthermore, Bayes' rule was utilized on that term to obtain the following equation:

(3) $$\begin{array}{c} P\left(x_{t}\right.\left|{\left\{y\right\}}_{1}^{t}\right)=\overset{M}{\underset{m=1}{\sum }}P\left(x_{t}\right.\left|{\left\{y\right\}}_{1}^{t},m\right)\frac{P\left({\left\{y\right\}}_{1}^{t}\left|m\right.\right)P\left(m\right)}{P\left({\left\{y\right\}}_{1}^{t}\right)}, \end{array}$$

where term *P*(*m*) is the probability of movement regime *m*. If the prior knowledge is not available, a uniform distribution is substituted. In order to calculate the posterior distribution recursively, one-step estimation is calculated as

(4) $$\begin{array}{c} P\left(x_{t}\right.\left|{\left\{y\right\}}_{1}^{t-1},m\right)=\int P\left(x_{t}\right.\left|x_{t-1},m\right)P\left(x_{t-1}\;|\;{\left\{y\right\}}_{1}^{t-1},m\right)dx_{t-1}. \end{array}$$

Then the posterior distribution conditioned on *m* can be obtained by Bayes' rule:

(5) $$\begin{array}{c} P\left(x_{t}\;|\;{\left\{y\right\}}_{1}^{t},m\right)=\frac{P\left(y_{t}\;|\;x_{t},m\right)P\left(x_{t}\;|\;{\left\{y\right\}}_{1}^{t-1},m\right)}{P\left(y_{t}\right.\left|{\left\{y\right\}}_{1}^{t-1},m\right)}, \end{array}$$

where the term *P*(**y**_*t*_|**x**_*t*_, {**y**}_1_^*t*−1^, *m*) has been replaced by *P*(**y**_*t*_|**x**_*t*_, *m*). Because given the current hand position and movement regime, the current neural activities are independent of neural activities from the beginning to last time step, as illustrated by the graphic model in Figure 2. We can calculate posterior distribution recursively by substituting (4) into (5) and feeding (5) back to (4) in the next time step. In practice, *P*(**x**_*t*_|{**y**}_1_^*t*^, *m*) was calculated by Gaussian approximation with parameters matched to the location and curvature of two terms [35]. The expectation and covariance matrix were calculated based on *P*(**x**_*t*_|{**y**}_1_^*t*^) to derive estimation and credible intervals.

### 2.4. Target Direction and Intended Movement Selection Prediction from Delay Epoch

Neural activities during planning (delay 1 and delay 2) contain key information for the forthcoming movement. In our application, there were two prior information, target direction and intended movement selection, which could be extracted in delay epoch (delay 1 and delay 2, resp.). Let *P*(*m*_1_|**y**_1_) be the estimation of target direction *m*_1_ given the neural activity **y**_1_ in delay 1, and *P*(*m*_2_|**y**_2_) be the estimation of intended movement selection *m*_2_ given the neural activity **y**_2_ in delay 2. With independence assumption, the estimation of final movement regimes *m* given the neural activity **y** in whole delay epoch can be calculated as

(6) $$\begin{array}{c} P\left(m\left|y\right.\right)=P\left(m_{1}\left|y_{1}\right.\right)P\left(m_{2}\left|y_{2}\right.\right). \end{array}$$

The estimation result of *P*(*m*|**y**) is substituted into the MTM as prior knowledge. In our study, two other estimations, *P*(*m*) and *P*(*m*_1_|**y**_1_), were also used to represent decoding with no prior and target direction prior only, respectively. The results were compared with *P*(*m*|**y**) substitution, which correspond to decoding with both target direction and intended movement selection prior.

To obtain the probabilities of movement regimes, statistical Bayes' rule was utilized in our study [16]. Supposing neural activities from all units are independent and the distribution of spikes for each movement regime is Gaussian [28, 36], the distribution of neural activity **y** from all units to each movement regime *m* can be fitted as follows:

(7) $$\begin{array}{c} y\left|m\right.\sim \overset{n}{\underset{i=1}{\prod }}N\left(y_{i};\mu_{i,m},\sigma_{i,m}^{2}\right), \end{array}$$

where *μ*_*i*,*m*_ and **σ**_*i*,*m*_^2^ are the mean and variance for the *i*th unit and *m*th movement regime and both of the parameters are obtained during training by maximum likelihood. For the test trials, the probability that movement regime is *m* conditioned on activity **y** in delay can be calculated by Bayes' rule illustrated in (4).

(8) $$\begin{array}{c} P\left(m\left|y\right.\right)=\frac{P\left(y\left|m\right.\right)P\left(m\right)}{P\left(y\right)}=\frac{P\left(y\left|m\right.\right)}{\sum_{m^{\prime }}P\left(y\left|m^{\prime }\right.\right)}, \end{array}$$

where *P*(*m*) is assumed to be uniform according to task settings. Actually, the accuracy of the estimated prior information is correlated with the duration and location of the time window, as well as the spike count model. Optimizing the prior information decoder is beyond the scope of our study. The time windows utilized to decode target direction and intended movement selection are shown in Figure 1(a).

## 3. Results

In this study, two monkeys (monkeys B and C) were well trained to perform the delayed obstacle-avoidance task. Accuracy rates of trials exceeded 95% and 93% for monkeys B and C, respectively. Neural signals in the PMd utilized in this study were recorded from 10 sessions (five for each monkey) distributed in one month, and each data session contained 307 ± 43 and 321 ± 50 trials for monkeys B and C. 45 ± 4.3 and 38 ± 3.8 units were isolated with Offline Sorter for monkeys B and C, respectively. Leave-one-out cross-validation was utilized in both target direction and movement selection prediction [18], which means one trial was regarded as a test sample and the rest trials were utilized to train the model parameters. Take one of the leave-one-out cases for example, the first to the last trials but one were training samples, and the last trial was the test sample.

### 3.1. Target Direction Encoding Properties in Delay 1 Epoch

We examined the target direction encoding properties during rest and delay 1 epoch. PV, which is defined as the summation of weighted preferred direction, was carried out to investigate the evolution of unit-encoding directions [15]. Velocity-based PV was utilized in our study [2]. Because all of the recorded units were analyzed regardless of their tuning depth, preferred direction was not normalized to unit [2]. So strong tuning had higher weights, and weak tuning had lower weights. Target direction was estimated from 0 to 900 ms (whole rest, delay 1 plus 100 ms in delay 2; considering causal time delay, the first bin in delay 2 was also collected for the prediction of target direction [37]), during which the moving cursor and target ball were shown to the monkeys.

**Figure 3:**
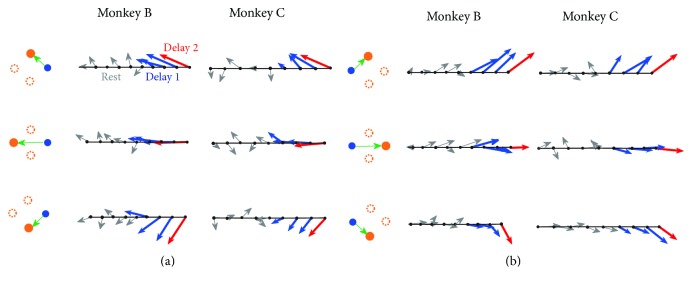
Temporal evolution of PVs across rest and delay epochs for both monkeys. (a) The specific task cases were shown on the left of each row, in which the arrow pointed from the start position (blue dot) to target (solid yellow dot). The dashed yellow circle represented other target candidates. The grey, blue, and red arrows represented PVs during rest, delay 1, and delay 2 epochs, respectively. The middle and right columns represented the PVs evolution for monkeys B and C, respectively. PV evolution in one row for a specific monkey represented the summation of PVs in 20 trials with the same task case for stability. (b) The same as (a) but with start point at the left. Trials were from sessions B140530 and C150430 for monkeys B and C, respectively.

Figure 3 demonstrates the behavior of velocity-based PVs for two starting position conditions for each animal as they changed over the course of rest and delay epochs. Five bins in rest, three bins in delay 1, and one bin in delay 2 were shown.  Figure 3(a) represents PV temporal evolution with start position on the right. During rest epoch with nothing on the screen, the direction of PVs remained insignificant. During delay 1 and first bin in delay 2, PVs pointed in the direction of the target with bigger length.  Figure 3(b) demonstrate the consistent results as (a) with the starting position on the left. Some PVs had direction preference during the rest epoch. For example, in cases of monkey B in Figure 3(b), PVs tended to point to the right during rest epoch and the angles between PVs and direct right were within 45 degrees. One possible reason is that an overtrained monkey could predict that the target would appear on the right (top right, bottom right, or direct right) during the trials, where the initial position was set on the left [38].

Bayes' rule with Gaussian hypothesis was utilized to estimate the target direction quantitatively. Neural activities labeled by red bars shown in Figure 1(b) were analyzed. Tables 1 and 2 demonstrate the expectation of target direction for monkey B and monkey C, respectively. The leave-one-out technique was utilized here to train Gaussian parameters. The accuracy rate was calculated as the expectation of selecting the right target. Student's *t*-test between the expectations and the chance level was performed.

### 3.2. Intended Movement Selection Encoding Properties in Delay 2 Epoch

**Figure 4:**
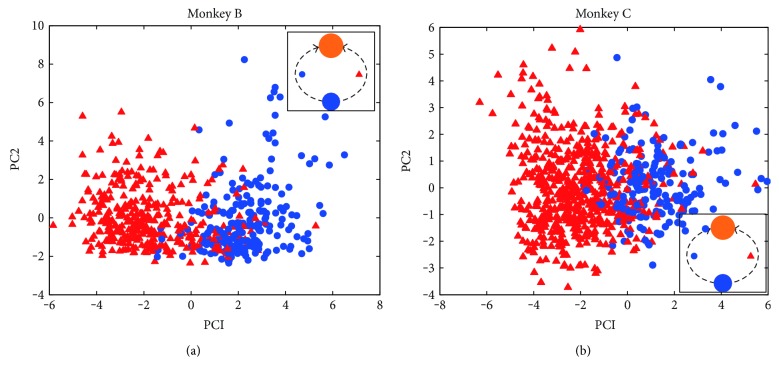
Low-dimension projections of neural activities during delay 2 for monkey B (a) and monkey C (b) in two representative sessions. (Inset) the two movement selections were represented by solid blue circles and red triangles, respectively. The *x*-axis and *y*-axis represented the first and second PC components, respectively. Trials were from sessions B140530 and C150430 for monkeys B and C, respectively.

Delay 2 epoch began with the appearance of the obstacle. There were two obstacle opening positions for each pair of start point and target. During delay 2 epoch with obstacle appearance, two intended movement selections (clockwise and counterclockwise) exist for monkeys to avoid the obstacle. We are interested in investigating whether there are differences in neural patterns between the two selections. Intended movement selection was estimated from 900 to 1300 ms (100–500 ms of delay 2), after the obstacle showed up. We use PCA to visualize neural patterns during delay 2 by dimensionality reduction, as shown in Figure 4. Each dot (blue circles and red triangles) in Figure 4 represents the neural pattern in 100 ms bin.  Figure 4(a) refers to PCA projection results for monkey B with start point at the bottom and target at the top. The opening positions of obstacles could appear on the left or right. The two obstacle-avoidance trajectory candidates were represented by dashed curves and labeled by solid circles and triangles. The neural activities projected to the top two PCA components were clustered into two groups with some overlaps, corresponding to trajectory candidates. Although there were some overlaps, the two clusters were distinguishable, which implies that monkeys were involved in the intended movement selection during delay 2 epoch.  Figure 4(b) shows the results of monkey C, which was consistent with (a).

We further utilized Bayes' rule with Gaussian hypothesis to estimate the intended movement selection. Neural activities during movement planning labeled by blue bars shown in Figure 1(b) were analyzed. Tables 3 and 4 demonstrate the expectation of intended movement selection for monkey B and monkey C, respectively. The leave-one-out technique was utilized here as well. Student's *t*-test between the expectations and the chance level was performed. Expectations of both monkeys were above the chance level significantly.

### 3.3. Decoding Results with Prior Knowledge

The prediction results of target direction and intended movement selection imply that the neural activities during delay epoch contained information of the task. We tried to integrate the predicted information during delay epoch to MTM framework to improve the trajectory estimation during movement. In this study, to evaluate the effects of prior knowledge on decoding performance in obstacle-avoidance task, decoders with three different prior knowledge were compared: (1) no prior knowledge (the prior term in RBE obeys uniform distribution); (2) integrating estimated target direction into RBE; and (3) integrating sequentially estimated target direction and intended movement selection into RBE.

**Figure 5:**
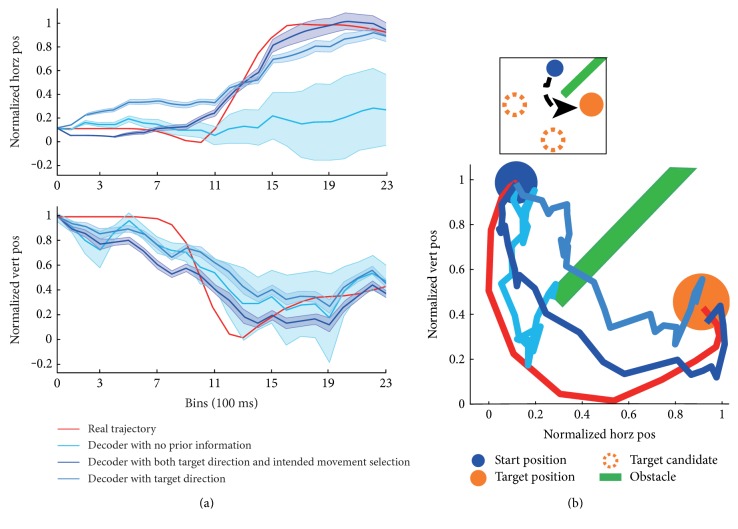
Decoding results with three decoders in one representative trial. (a) Normalized horizontal and vertical trajectories in one trial. The *x*-axis represents the samples (each sample has time windows of 100 ms) and normalized kinematics. The red line represents the real trajectory. The lines with light blue, blue, and dark blue mean estimation with no prior, target direction only, and target and intended movement selection, respectively. The shadows around the lines represent 95% credible intervals. (b) The reconstructed 2D reaching trajectories. The inset at the top represents the representative trajectory in the workspace. The small blue and big yellow ball represent the start position and target position, respectively. The dashed yellow circles represent the target candidates. The green bar represents the obstacle. The *x*-axis and *y*-axis represent normalized horizontal and vertical positions, respectively.

Figure 5(a) shows decoding results in horizontal and vertical positions, respectively, while Figure 5(b) demonstrates the estimated trajectories in two-dimensional space. Decoder with no prior knowledge performed worst with the largest 95% credible interval, as illustrated in Figure 5(a). The estimated trajectory followed the real trajectory relatively well for the first half, which may even be steered by the obstacle. However, it lost direction in the second half and failed to reach the target. The estimation with target direction only tended to reach the target in a relatively direct way, which may run into the obstacle. The trajectory estimated with both target direction and intended movement selection curved to steer around the obstacle and reached the target successfully, as shown in Figure 5(b).

**Figure 6:**
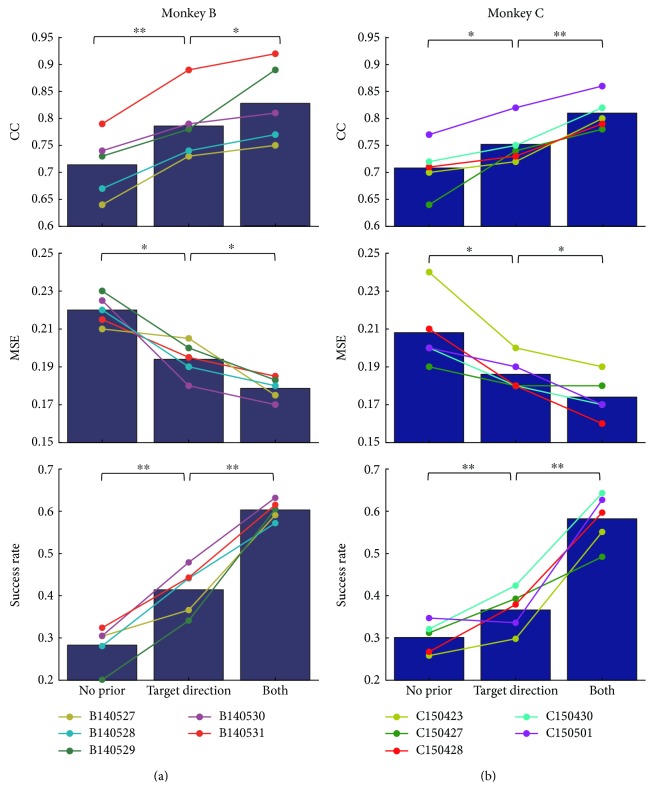
Summary of estimation performance with different decoders for monkey B (a) and monkey C (b). Decoding performances with no prior, target direction only, and both target direction and intended movement selection were shown. The *x*-axis represents three decoders, and the *y*-axis represents the decoding performance (CC, MSE, and success rate). Dots with different colors represent the mean decoding performance for each data session. Dots in one line represent performances with different prior knowledge for one data session. Significance level (paired Student's *t*-test) of 0.05 (∗) and 0.01 (∗∗) was shown.

Pearson's correlation coefficient (CC) and mean square error (MSE) were utilized to evaluate the performance of trajectory regression. Success rate means the rate of trials that monkeys steered around the obstacles and reached the target successfully, which was utilized to evaluate the decoding performance in view of task completion.  Figure 6 shows estimation performance of three decoders with different prior knowledge for both monkeys across ten data sessions.  Figure 6(a) shows the mean CC (top), MSE (middle), and success rate (bottom) of each data session labeled by different colors for monkey B. The histograms represent the mean CC, MSE, and success rate of all the data sessions. Histograms show that decoder with no prior information had the smallest CC and biggest MSE, while decoder with both target direction and intended movement selection had the biggest CC and smallest MSE. Actually, the CC of the decoder with both target direction and intended movement selection exceeded that without prior information, with only target direction by 15.9% and 5.3%, respectively (MSE descending rate: 18.8%, 7.9%).  Figure 6(b) shows the decoding performance of monkey C (CC ascending rate: 14.4%, 7.7%; MSE descending rate: 16.4%, 6.5%), which was consistent with the results shown in (a). The success rate obtained by both target direction and intended movement selection exceeded that without prior information, with only target direction by 113.1% and 45.7% for monkey B and 93.4% and 59.0% for monkey C, respectively. With more prior knowledge, decoders obtained more instructive information, which improved the trajectory estimation and trial completion performance. More information was needed to estimate the trajectory in a complex task. The results demonstrate that both target direction and intended movement selection were essential in the obstacle-avoidance task.

## 4. Discussion and Conclusion

In this study, we predicted the target direction and intended movement selection during delay epoch and integrated the planning information to MTM framework to improve the decoding performance in movement epoch. The results of PV and PCA demonstrated that units tuned to the target direction and initial movement direction during delay 1 and delay 2, respectively. We sequentially integrated this two prior knowledge to MTM. Compared to the decoders with no prior and only estimated target direction, the CC of trajectory estimation was promoted by 15.9% and 5.3%, and 14.4% and 7.7% for monkeys B and C, respectively, while the descending rates of MSE were 18.8% and 7.9%, and 16.4% and 6.5% for monkeys B and C, respectively. The trial success rates were improved significantly with both target direction and intended movement selection for both monkeys. Results imply that integrating target direction and intended movement selection could improve the hand trajectories estimation in an indirect reaching.

The indirect reaching is common in daily life. The environment animals live in is very complex and full of obstacles, which poses difficulties for decoding. The study here proposes an approach to generalize the BMIs from a point-to-point task to more complex task with planning information integration strategy. The PMd is considered to be related to planning during delay epoch [21–23]. Pearce and Moran have visualized the planning activities of the PMd by PVs [2]. And the evolution of PVs during delay 1 epoch in this study agreed with the above report. We also found that the neural activities during delay 2 tuned to the intended movement selection. The neural activities during rest epoch of some trials were beyond our expectation. We found that both monkeys made some prejudging during the rest epoch based on the task settings. That implies that to some extent, overtrained monkeys had the sense of workspace and made the prejudging based on the hand position [38]. Furthermore, the shape or the place of the obstacle might influence the performance of the trajectory estimation. Although our study mainly focused on incorporating prior information to improve decoding performance, it would be important to further study the influence of obstacles by using indirect reach movements in the following studies.

MTM framework was proposed to improve the trajectory estimation by integrating target information [16]. This framework works well by conforming to the timeline of performing a task: first planning and then moving. For some more complex tasks, neural activities during planning are always corresponding to the key information about the task. So the extracted planning information provide some instructions for the following estimation. In this study, we generalize this framework to a more complex task by integrating one more prior knowledge. It is easy to extend the framework to three or more prior knowledge by our methods. With more prior information included, the trajectory of more complex tasks could be estimated smoothly and accurately. As mentioned in the Introduction, the state-of-the-art ReFIT-KF promotes the online reaching movement estimation performance by retraining the parameters with the intention to target information [3]. The comparison of ReFIT-KF and methods here would be conducted in online BMIs in further study.

There are some limitations in our study. In practice, the situation is more complex than the task performed in our study. So more complex models [39] should be utilized to extract more valid information. In addition, the time windows to extract the planning information were fixed in this study, where uncorrelated neural activities were involved in. Several methods have been proposed to estimate the state evolution during the task [39–42]. However, detecting the time windows that planning happens is still an open question. Only offline analysis was carried out here. More experiments for online validation [17] needs to be done in the following studies.

## Figures and Tables

**Table 1:** Expectation of target direction prediction for monkey B. Asterisks represent the significance level (Student's *t*-test) of 0.05 (∗) and 0.01 (∗∗) that the expectations were above chance level (0.33).

Data sessions for monkey B	Expectation of target direction prediction
B140527	0.43 ± 0.13^∗^
B140528	0.42 ± 0.12^∗^
B140529	0.42 ± 0.09^∗∗^
B140530	0.58 ± 0.12^∗∗^
B140531	0.56 ± 0.13^∗∗^

**Table 2:** Expectation of target direction prediction for monkey C. Asterisks represent the significance level (Student's *t*-test) of 0.05 (∗) and 0.01 (∗∗) that the expectations were above chance level (0.33).

Data sessions for monkey C	Expectation of target direction prediction
C150423	0.52 ± 0.13^∗^
C150427	0.51 ± 0.11^∗^
C150428	0.48 ± 0.09^∗∗^
C150430	0.58 ± 0.12^∗∗^
C150501	0.74 ± 0.11^∗∗^

**Table 3:** Expectation of intended movement selection prediction for monkey B. Asterisks represent the significance level (Student's *t*-test) of 0.05 (∗) and 0.01 (∗∗) that the expectations were above chance level (0.5).

Data sessions for monkey B	Expectation of intended movement selection prediction
B140527	0.64 ± 0.12^∗^
B140528	0.57 ± 0.13^∗^
B140529	0.72 ± 0.10^∗∗^
B140530	0.70 ± 0.11^∗∗^
B140531	0.63 ± 0.12^∗^

**Table 4:** Expectation of intended movement selection prediction for monkey C. Asterisks represent the significance level (Student's *t*-test) of 0.05 (∗) and 0.01 (∗∗) that the expectations were above chance level (0.5).

Data sessions for monkey C	Expectation of intended movement selection prediction
C150423	0.74 ± 0.11^∗∗^
C150427	0.69 ± 0.11^∗∗^
C150428	0.62 ± 0.10^∗^
C150430	0.68 ± 0.12^∗^
C150501	0.59 ± 0.13^∗^

## References

[1] Hochberg L. R., Bacher D., Jarosiewicz B. (2012). Reach and grasp by people with tetraplegia using a neurally controlled robotic arm. *Nature*.

[2] Pearce T. M., Moran D. W. (2012). Strategy-dependent encoding of planned arm movements in the dorsal premotor cortex. *Science*.

[3] Gilja V., Nuyujukian P., Chestek C. A. (2012). A high-performance neural prosthesis enabled by control algorithm design. *Nature Neuroscience*.

[4] Welch G., Bishop G. (2006). *An Introduction to the Kalman Filter*.

[5] Zhang Q., Zhang S., Hao Y. (2012). Development of an invasive brain-machine interface with a monkey model. *Chinese Science Bulletin*.

[6] Roelfsema P. R., Ooyen A. V. (2005). Attention-gated reinforcement learning of internal representations for classification. *Neural Computation*.

[7] Wang Y., Wang F., Xu K., Zhang Q., Zhang S., Zheng X. (2015). Neural control of a tracking task via attention-gated reinforcement learning for brain-machine interfaces. *IEEE Transactions on Neural Systems and Rehabilitation Engineering*.

[8] Wessberg J., Stambaugh C. R., Kralik J. D., Beck P. D., Laubach M. (2000). Real-time prediction of hand trajectory by ensembles of cortical neurons in primates. *Nature*.

[9] Carmena J. M., Lebedev M. A., Crist R. E. (2003). Learning to control a brain-machine interface for reaching and grasping by primates. *PLoS Biology*.

[10] Velliste M., Perel S., Spalding M. C., Whitford A. S., Schwartz A. B. (2008). Cortical control of a prosthetic arm for self-feeding. *Neurosurgery*.

[11] Taylor D. M., Tillery S. I., Schwartz A. B. (2002). Direct cortical control of 3D neuroprosthetic devices. *Science*.

[12] Serruya M. D., Hatsopoulos N. G., Paninski L., Fellows M. R., Donoghue J. P. (2002). Brain-machine interface: instant neural control of a movement signal. *Nature*.

[13] Georgopoulos A. P., Kalaska J. F., Caminiti R., Massey J. T. (1982). On the relations between the direction of two-dimentional arm movements and cell discharge in primate motor cortex. *The Journal of Neuroscience*.

[14] Weinrich M., Wise S. P. (1982). The premotor cortex of the monkey. *Journal of Neuroscience*.

[15] Georgopoulos A., Schwartz A., Kettner R. (1986). Neuronal population coding of movement direction. *Science*.

[16] Yu B. M., Kemere C., Santhanam G. (2007). Mixture of trajectory models for neural decoding of goal-directed movements. *Journal of Neurophysiology*.

[17] Bishop W., Yu B. M., Santhanam G. (2008). The use of a virtual integration environment for the real-time implementation of neural decode algorithms. *Conference Proceedings: Annual International Conference of the IEEE Engineering in Medicine and Biology Society*.

[18] Shanechi M. M., Williams Z. M., Wornell G. W., Hu R. C., Powers M., Brown E. N. (2013). A real-time brain-machine interface combining motor target and trajectory intent using an optimal feedback control design. *PloS One*.

[19] Sadtler P. T., Ryu S. I., Tylerkabara E. C., Yu B. M., Batista A. P. (2015). Brain-computer interface control along instructed paths. *Journal of Neural Engineering*.

[20] Hocherman S., Wise S. P. (1991). Effects of hand movement path on motor cortical activity in awake, behaving rhesus monkeys. *Experimental Brain Research*.

[21] Wise S. P. (1985). The primate premotor cortex: past, present, and preparatory. *Annual Review of Neuroscience (Palo Alto, CA)*.

[22] Cisek P., Kalaska J. F. (2005). Neural correlates of reaching decisions in dorsal premotor cortex: specification of multiple direction choices and final selection of action. *Neuron*.

[23] Churchland M. M. (2006). Neural variability in premotor cortex provides a signature of motor preparation. *Journal of Neuroscience*.

[24] Shanechi M. M., Orsborn A. L., Moorman H. G., Gowda S., Dangi S., Carmena J. M. (2017). Rapid control and feedback rates enhance neuroprosthetic control. *Nature Communications*.

[25] Li H., Wang F., Zhang Q., Principe J. C. Maximum correntropy based attention-gated reinforcement learning designed for brain machine interface.

[26] Hotson G., Smith R. J., Rouse A. G., Schieber M. H., Thakor N. V., Wester B. A. (2016). High precision neural decoding of complex movement trajectories using recursive Bayesian estimation with dynamic movement primitives. *IEEE Robotics and Automation Letters*.

[27] Brown E. N., Frank L. M., Tang D., Quirk M. C., Wilson M. A. (1998). A statistical paradigm for neural spike train decoding applied to position prediction from ensemble firing patterns of rat hippocampal place cells. *The Journal of Neuroscience*.

[28] Yu B. M., Ryu S. I., Santhanam G., Churchland M. M., Shenoy K. V. (2004). Improving neural prosthetic system performance by combining plan and peri-movement activity. *Conference Proceedings: Annual International Conference of the IEEE Engineering in Medicine and Biology Society*.

[29] Kemere C., Santhanam G., Yu B. M., Ryu S., Meng T., Shenoy K. V. (2004). Model-based decoding of reaching movements for prosthetic systems. *Conference Proceedings: Annual International Conference of the IEEE Engineering in Medicine and Biology Society*.

[30] Brockwell A. E., Rojas A. L., Kass R. E. (2004). Recursive Bayesian decoding of motor cortical signals by particle filtering. *Journal of Neurophysiology*.

[31] Wu W., Gao Y., Bienenstock E., Donoghue J. P., Black M. J. (2006). Bayesian population decoding of motor cortical activity using a Kalman filter. *Neural Computation*.

[32] Shanechi M. M., Wornell G. W., Williams Z. M., Brown E. N. (2013). Feedback-controlled parallel point process filter for estimation of goal-directed movements from neural signals. *IEEE Transactions on Neural Systems and Rehabilitation Engineering*.

[33] Maynard E. M., Nordhausen C. T., Normann R. A. (1997). The Utah intracortical electrode array: a recording structure for potential brain-computer interfaces. *Electroencephalography and Clinical Neurophysiology*.

[34] Chhatbar P. Y., von Kraus L. M., Semework M., Francis J. T. (2010). A bio-friendly and economical technique for chronic implantation of multiple microelectrode arrays. *Journal of Neuroscience Methods*.

[35] MacKay D. J. C. (2003). *Information Theory, Inference, and Learning Algorithms*.

[36] Maynard E. M., Hatsopoulos N. G., Ojakangas C. L. (1999). Neuronal interactions improve cortical population coding of movement direction. *Journal of Neuroscience*.

[37] Moran D. W., Schwartz A. B. (1999). Motor cortical representation of speed and direction during reaching. *Journal of Neurophysiology*.

[38] Hoshi E., Tanji J. (2000). Integration of target and body-part information in the premotor cortex when planning action. *Nature*.

[39] Kao J. C., Nuyujukian P., Ryu S. I., Shenoy K. V. (2015). A high-performance neural prosthesis incorporating discrete state selection with hidden Markov models. *IEEE Transactions on Biomedical Engineering*.

[40] Aggarwal V., Mollazadeh M., Davidson A. G., Schieber M. H., Thakor A. N. V. (2013). State-based decoding of hand and finger kinematics using neuronal ensemble and LFP activity during dexterous reach-to-grasp movements. *Journal of Neurophysiology*.

[41] Kemere C., Santhanam G., Yu B. M. (2008). Detecting neural-state transitions using hidden Markov models for motor cortical prostheses. *Journal of Neurophysiology*.

[42] Achtman N., Afshar A., Santhanam G., Yu B. M., Ryu S. I., Shenoy K. V. (2007). Free-paced high-performance brain-computer interfaces. *Journal of Neural Engineering*.

